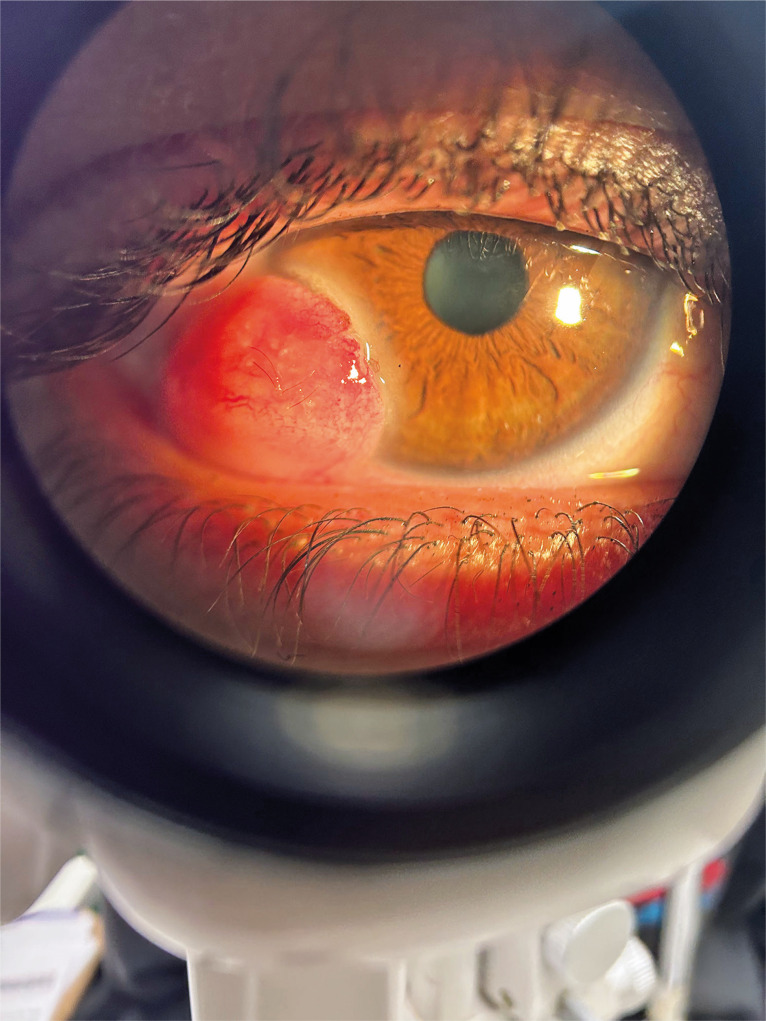# Corneal dermoid

**DOI:** 10.5935/0004-2749.2024-0405

**Published:** 2025-02-13

**Authors:** Amanda Ginelli, Nicole Bulgarão Maricondi de Almeida, Newton Kara-Júnior

**Affiliations:** 1 Ophthalmology department, Hospital das Clínicas, Universidade de São Paulo, São Paulo, SP, Brazil.

Corneal dermoid (Figure) is a congenital benign tumor and ocular malformation^(1)^ generally classified into three grades of severity^(2)^, with the most common site being the right eye’s temporal limbus^(1)^. Corneal dermoid can result in visual impairment, restricted eye movement, and facial asymmetry^(1)^. Pathological features include squamous epithelium coverage, hair follicles, sebaceous glands, and adipose and fibrous tissue^(1)^. Surgery is effective^(1)^, although complications include persistent epithelial defects and peripheral corneal vas-cularization and opacity^(2)^.

**Figure 1:**